# ClC Chloride Channels in Gram-Negative Bacteria and Its Role in the Acid Resistance Systems

**DOI:** 10.4014/jmb.2303.03009

**Published:** 2023-04-14

**Authors:** Minjeong Kim, Nakjun Choi, Eunna Choi, Eun-Jin Lee

**Affiliations:** Department of Life Sciences, College of Life Sciences and Biotechnology, Korea University, Seoul 02841, Republic of Korea

**Keywords:** *clcA*, acid resistance, ClC channel, chloride antiporter

## Abstract

Pathogenic bacteria that colonize the human intestinal tract have evolved strategies to overcome acidic conditions when they pass through the gastrointestinal tract. Amino acid-mediated acid resistance systems are effective survival strategies in a stomach that is full of amino acid substrate. The amino acid antiporter, amino acid decarboxylase, and ClC chloride antiporter are all engaged in these systems, and each one plays a role in protecting against or adapting to the acidic environment. The ClC chloride antiporter, a member of the ClC channel family, eliminates negatively charged intracellular chloride ions to avoid inner membrane hyperpolarization as an electrical shunt of the acid resistance system. In this review, we will discuss the structure and function of the prokaryotic ClC chloride antiporter of amino acid-mediated acid resistance system.

## Introduction

Bacterial pathogens that colonize the gut, such as *Salmonella enterica*, *Escherichia coli*, *Vibrio cholerae*, and *Listeria monocytogenes*, invade the human intestine via the oral tract and stomach. When these bacteria pass through the gastrointestinal tract, they are exposed to a wide range of pH. These bacteria experience severely acidic environments lower than pH 2.0, particularly in the stomach [1]. However, because most neutralophiles like *E. coli* maintain their internal pH homeostasis around 7.2 to 7.8, there is a significant difference between the internal pH and the external acidic environment [2]. Bacteria have developed resistance mechanisms to deal with different types of acidic environments in the gastrointestinal tract.

The extreme acid resistance (XAR) system enables them to withstand extremely acidic environments less than pH 3, the lowest pH value of an empty stomach [3]. When the pH is between 5.5 and 6, they adjust to nonlethal mildly acidic environments by using the acid tolerance response (ATR) mechanism [4]. No requirement of an adaptive response at the mild acidic pH distinguishes XAR from ATR [5]. ATR focuses on maintaining intracellular homeostasis against acid stress and, additionally, it provides tolerance to extremely acidic environments (< pH 3.0) when it is pre-adapted in mild acidic conditions. On the other hand, XAR directly defends against severe acid that reduces cell viability due to the change of intracellular pH. It is currently understood that bacteria modify the intracellular pH in response to externally acidic conditions through five distinct AR systems including ATR and XAR, to help bacteria increase survival [6].

**Fig. 1:**
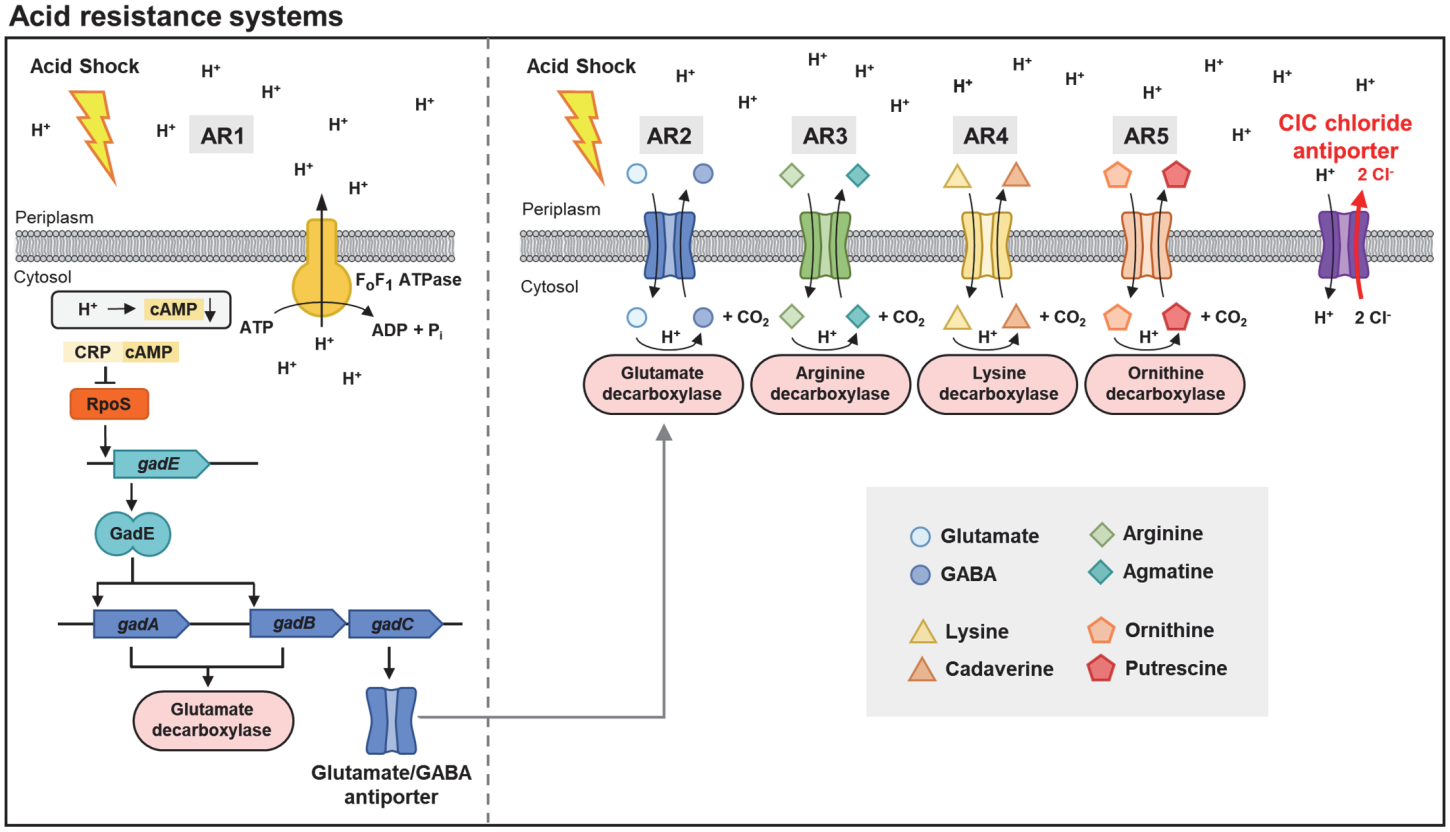
The prokaryotic acid resistance systems. Acid resistance systems in gram-negative bacteria (AR1-5). AR1 requires F_o_F_1_ ATPase for acid resistance. When bacteria are challenged by extremely acidic conditions, levels of cyclic adenosine monophosphate (cAMP) drop, which otherwise bind to the receptor (CRP) and inhibit synthesis of RpoS. The decrease in cAMP levels thus enhances RpoS and promotes production of GadE. And, GadE enhances transcription of *gadABC* to control AR2. The right side panel represents the amino acid-dependent acid resistance systems including AR2, AR3, AR4, and AR5. The substrates enter through amino acid-product antiporters and are decarboxylated via the respective decarboxylases into positively charged products, which are then exported through the antiporters. During decarboxylation, protons are consumed by decarboxylases. Chloride ions, the counterions of hydrochloric acid, are exported by ClC chloride antiporters. The substrates and products are indicated on the bottom side of the panel.

All five AR systems are induced at stationary phase, but they can be divided into two groups. Utilizing particular amino acids as substrates to increase bacteria's internal pH serves as the criterion for dividing these groups. First, AR1 is the only system that does not utilize amino acids [7]. For acid resistance, the AR1 utilizes alternative sigma factor σ^s^ and cAMP receptor protein (CRP) [8, 9]. RpoS sigma factor contributes to AR1 by controlling transcription of acid resistance genes, while CRP and cyclic AMP control synthesis of RpoS sigma factor [10]. ClC chloride channels are not necessary in this system (Fig. 1).

AR2, AR3, AR4, and AR5 are acid resistance systems mediated by amino acids that employ similar processes [8, 11, 12]. These four AR systems (AR2, AR3, AR4, and AR5) use glutamate, arginine, lysine, and ornithine as substrates, respectively, and the substrates enter the cell through antiporters if they are present in the extracellular solution (Fig. 1) [13]. Imported glutamate, arginine, lysine, and ornithine are used to produce γ-aminobutyrate (GABA), agmatine, cadaverine, and putrescine by respective decarboxylases, which are then released back to the periplasmic space by antiporters (Table 1). The amino acid decarboxylases continuously consume protons in order to remove the carboxyl group from amino acids and attach a positive hydrogen ion. Chloride ions, counterions of protons in hydrochloric acid, are exported through the ClC chloride channel. Elimination of chloride ions by the ClC channel prevents the accumulation of negatively charged chloride ions in the cytoplasm and the hyperpolarization of inner membrane [14].

To reduce the internal proton concentration, amino acid-mediated AR systems utilize intracellular protons while simultaneously decarboxylating substrates. They aid to maintain pH homeostasis and internal pH, which promotes cell survival [15]. Since *Salmonella* cannot survive with an internal pH of less than 5.5, these mechanisms are advantageous in increasing both the internal and external pH [16].

In extremely acidic conditions, the intracellular proton concentrations continuously increase due to the influx of HCl and the activity of ClC chloride channels. Bacterial cells must therefore evaluate how they consume or release these internal protons. For glucose-dependent AR1, the F_o_F_1_ H^+^-translocating ATPase on the cell membrane causes the release of intracellular protons into the extracellular environment [17]. As a result, if the F_o_F_1_ ATPase is defective, AR1 cannot function properly, whereas AR2 and 3 are unaffected regardless of the presence of ATPase [15]. During acid stress at pH 2.5, both AR2 and AR3 elevate the intercellular pH [15]. Moreover, at pH 2.5, the electrochemical gradient (Δψ) across the membrane was reversed by AR2 and AR3 to survive in the extremely acidic conditions. Reversal of membrane potential from inside negative to inside positive charge might be beneficial for *E. coli* to minimize excessive proton motive force generated during acid stress [15, 18, 19]. Simultaneously, the ClC channel exchanges internal protons with external chloride ions in opposite directions in order to balance positive electrical potential [20].

On the other hand, bacteria including *E. coli* convert unsaturated fatty acids into cyclopropane fatty acids, which are in part controlled by RpoS [21]. Because the unsaturated fatty acids contribute to internal membrane fluidity, decreasing membrane proton permeability prevents the accumulation of intracellular protons ahead of time. In conclusion, bacteria have both resistance and protection strategies against extremely acidic stress, which are similar to those of acidophiles thriving under acidic conditions.

## The Conserved ClC Channel Family in Gram-Negative Bacteria

**Fig. 2:**
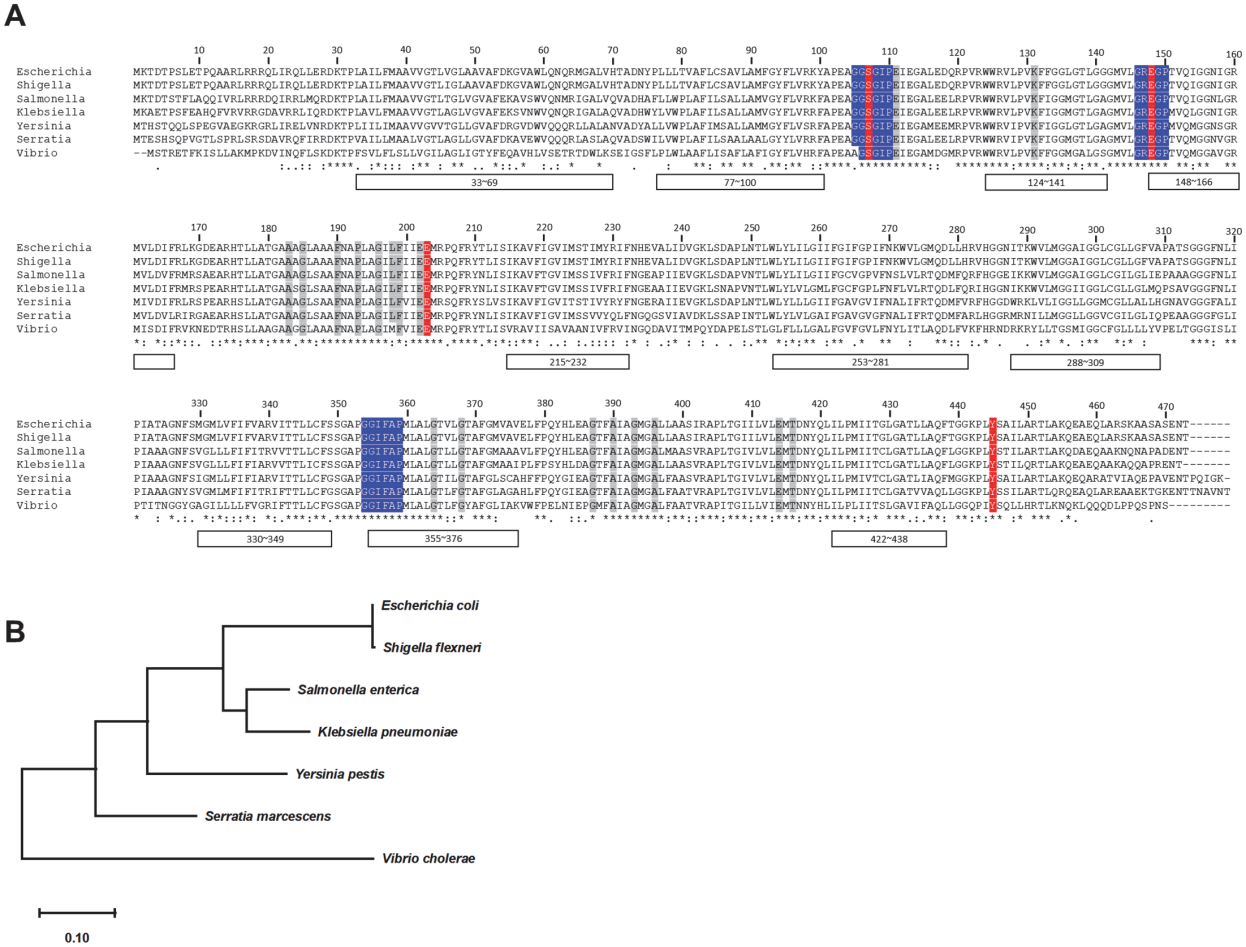
Sequence alignment of ClC family antiporters in gram-negative bacteria. (A) Amino acid sequence alignment of ClcA in *Escherichia coli* (P37019), *Shigella flexneri* (A0A658YVD4), *Salmonella* typhimurium (E8XI01), *Klebsiella pneumoniae* (A0A2A2SXY4), *Yersinia pestis* (Q8ZBM0), *Serratia marcescens* (A0A656VRJ7), and *Vibrio cholerae* (A0A8G0CF81). Transmembrane helices are indicated as boxes with numbering (*E. coli* K-12). Key residues involved in the chloride ion binding and transport in the ClC chloride antiporter are highlighted in red. Residues involved in the ion selectivity filter are blue. Conserved sequences within the ClC family including eukaryotes are indicated in grey. Asterisks indicate positions that are fully conserved, colons indicate strong conservations (>0.5), and periods indicate weak conservations (= or < 0.5). The sequence alignment was analyzed by CLUSTALW. (**B**) Phylogenetic tree of ClcA proteins from the species listed in (**A**). Phylogenetic tree are visualized using NCBI Tree Viewer; the branch lengths are displayed at the bottom.

The ClC chloride channel family is a conserved protein group that is widely distributed from prokaryotes to human muscle cells [22]. Yet, the sequence similarities between kingdoms are quite low, ranging from 15 to 20% [23]. Although overall similarities are low, chloride coordinating “hot spots” and key residues are highly conserved across the species and kingdoms (Fig. 2A) [24]. Furthermore, the hydrophobicity patterns are strongly conserved in all known ClC channels. These ClC channel proteins share nine to twelve transmembrane helices and membrane topology [25, 26].

In the phylogenetic tree of ClcA, *Shigella flexneri* is close to *E. coli*, which is expected to possess a similar XAR mechanism (Fig. 2B) [6, 27]. However, despite sharing the sequence similarity, *V. cholerae* is very different from the other species on the ClcA phylogenetic tree. This might be due to that *V. cholerae* lacks XAR system and hence only a small number of bacteria survive at pH 2.5, making them less likely to colonize the intestine [5].

## Functional Characterization of ClcA in the Amino Acid-Mediated AR System

In the amino acid-mediated AR systems, the ClC chloride channels exchange two internal chloride ions for one proton from the outside of the cell [28]. This transporter does more than just release excess ions in the electrogenic process. Because the amino acid antiporters export more positively charged products than the substrates (for examples, Glu to GABA^+^ / Arg^+^ to Agm^2^+), transmembrane potential is shifted to inside negative. At the same time, chloride ions accumulate in the cytoplasm due to HCl ionization, resulting in inner membrane hyperpolarization [14]. In these circumstances, the ClC channels are used as electric shunts to counteract hyperpolarization.

The primary gene of the prokaryotic ClC channel family, *clcA* (also known as *eriC* or *yadQ*), controls the amino acid-mediated AR system to be functional, which is linked to cell survival [29]. Despite the fact that there is no impairment depending on the number or function of amino acid-substrate antiporters or amino acid decarboxylases, the rate of releasing decarboxylated products to extracellular solution from *E. coli* with a defective ClC channel is significantly lower than that of wild-type [14]. In the case of *V. cholerae*, the survival rate of the *clcA* knock-out strain decreased over time at pH 5 [30]. According to these findings, the prokaryotic ClC channel *clcA* gene controls the entire amino acid-mediated AR systems, and it affects the survival of bacterial pathogens in a wide range of acidic environments.

Because ClcA influences the AR systems, its activity is dramatically reduced when the AR system is not required, such as neutral or basic pH conditions [14]. A *V. cholerae* strain deleting ClC channel showed sensitivity to acidic stress but unexpectedly exhibited increased intestinal colonization in infant mice model compared to control group [31]. Later, a search for *in vivo* repressed genes using a recombination-based *in vivo* expression technology (RIVET) implicates that *clcA* gene is required for acidic resistance in the stomach but its expression needs to be repressed in the lower gastrointestinal tract because its presence in the lower intestinal tract decreases *V. cholerae* survival fitness in infant mouse model [30].

## Structure and Function in Prokaryotic ClC Chloride Antiporter

The ClC subclasses of the ClC family are split into two categories: transmembrane ion channels and antiporters [25]. The ClC channels and transporters have a similar overall structure [23]. Among them, prokaryotic ClC is an H^+^/Cl- antiporter that exports chloride ions outside of gram-negative bacteria such as *E. coli*, S. Typhimurium, and *V. cholerae*.

Prokaryotic ClC chloride antiporter is a double-pore homodimer that is fundamentally different from a common cation channel, which is usually a single-pore protein with four- or five-fold symmetry [32, 33]. In the case of S. Typhimurium, ClC antiporter has 18 helices, the majority of which are tilted and embedded in the membrane [34]. Due to its symmetry, each subunit's pore is twisted rather than aligned straight [35]. Also, the pore lining is composed of four sequence segments [36].

The prokaryotic ClC chloride antiporter undergoes distinctive conformational changes during Cl- transport cycle, releasing two chloride ions and one proton on opposite direction [37]. Chloride antiporter alternates opening and closing of two pores in the extracellular and intracellular sides in concert with the conformational changes. Major steps are following: 1) Two pores open outwards and bind to chloride ions, but both the intracellular pores are closed. 2) Two pores open inwards and release chloride ions, but extracellular pores are closed. At this time, key residues (E203 in *E. coli*) for proton transport face cytoplasm and accessible for proton transport. In between these two steps, there are transitory conformational changes toward inward or outward opening of the two pores.

**Fig. 3:**
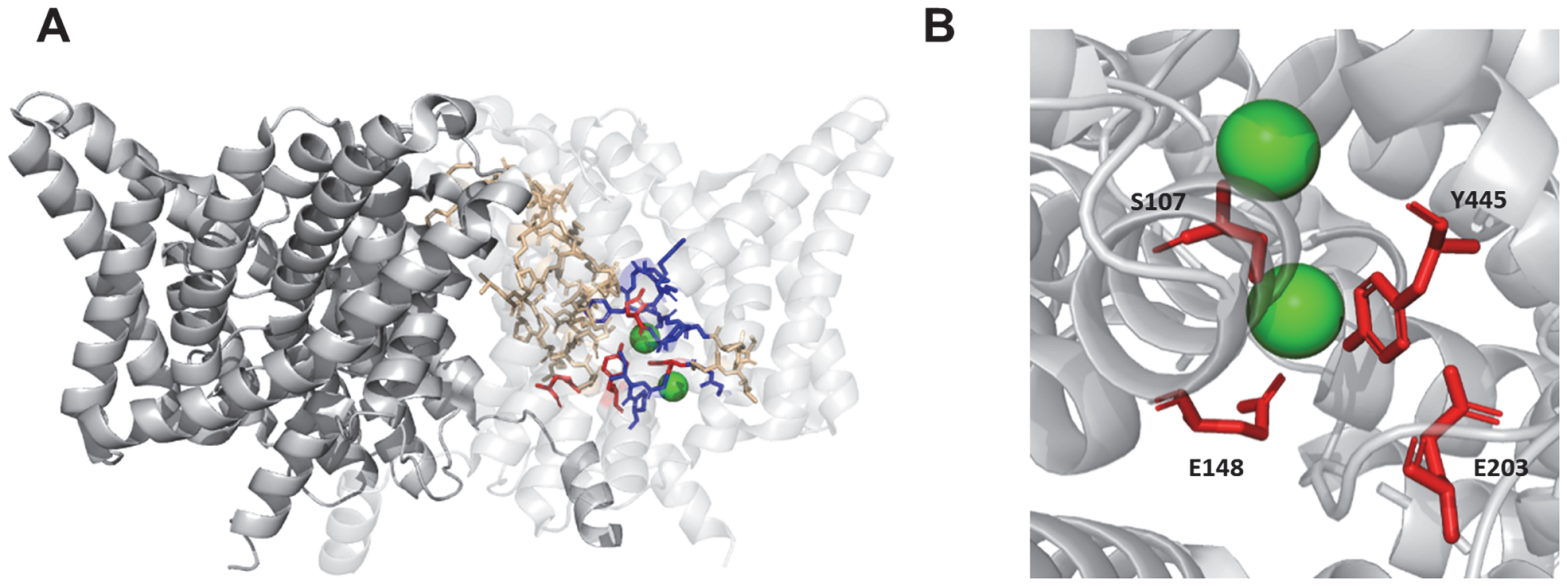
Structure of ClcA homodimer. (A) An overall structure of ClcA homodimer (PDB 1OTS). Yellow-colored residues are conserved regions of ClcA sequences in Fig. 2. Red-colored residues (S107, E148, E203, and Y445) are key residues in the selectivity filter and chloride ion binding in ClcA. Blue-colored residues are additional selectivity filter in ClcA. (**B**) A magnified view of four key residues involved in glutamate gates and chloride ion binding. Green spheres represent chloride ions in the ClC transporter. The homodimer is visualized using PyMOL. To distinguish each monomer, one subunit of the homodimer is visualized more transparently.

Key residues were identified in crystallographic studies of ClC antiporter, which are widely conserved and function as selectivity filters by coordinating chloride ion and proton (Fig. 3) [38]. Glutamate side chains directly control the passage of chloride ion depending proton binding status, thus coupling chloride ion movement to proton transport. E148 residue functions as a gate by blocking the movement of chloride ions in a deprotonated state but it also involves the release of chloride ions in a protonated state [39, 40]. In addition to E148, E203 residue affects chloride-proton antiport via protonation or deprotonation in the transmembrane region [41]. When the extracellular pores open and E148 is protonated, E203 is buried in the protein interior and inhibits protonation [33]. Although E148 and E203 participate in the proton transport pathway, the distance between the two residues is as far as 15 Å, which is not enough to transport the proton with a single movement [42]. For that reason, the hydroxyl group of Y445, which is located between two glutamates, directly coordinates with the central chloride ion in the ClC antiporter. Because the electrophilic aromatic ring of Y445 serves as a proton donor, chloride ions are easily accessible [34]. Mutations in Y445 render the protein inactive or weaken proton coupling, resulting in a slower rate of proton transport [33, 43].

There are four key residues (S107, E148, E203, and Y445) that directly coordinate chloride ion transport (Fig. 3B). S107, similarly to Y445, coordinates with oxygen in the amino acid side-chain and directly binds to chloride ions, affecting ion conductance in the pore [34]. When bound to the polar functional group, chloride ions interact with additional hydrophobic amino acid side chains and are released into the extracellular space, resulting in conformational change.

## Another ClC Channel Family Protein, ClcB

**Fig. 4:**
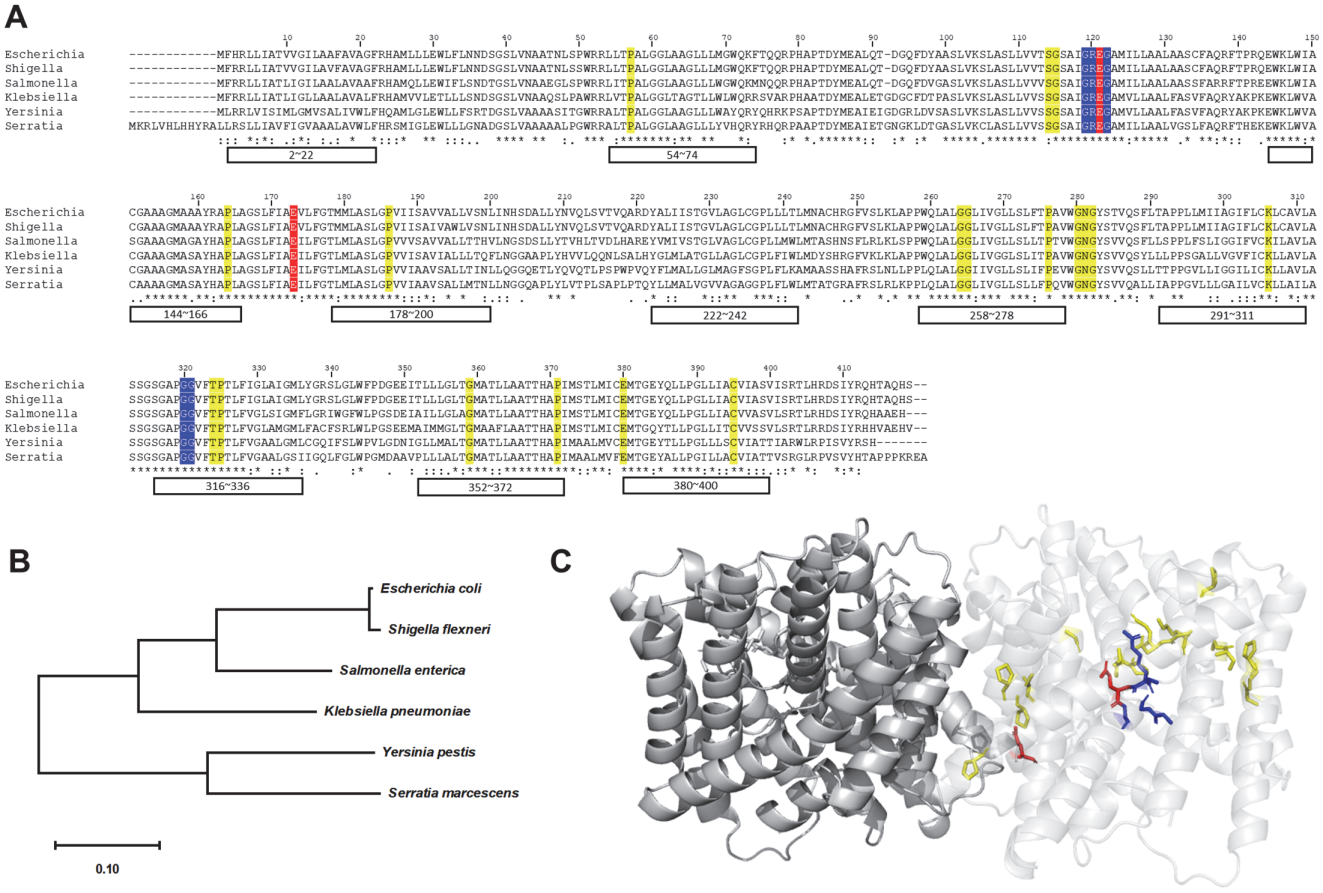
Amino acid alignment and structures of ClcB homodimer. (**A**) Amino acid sequence alignment of ClcB proteins from *Escherichia coli* (P76175), *Shigella flexneri* (P59638), *Salmonella* typhimurium (Q8ZPK5), *Klebsiella pneumoniae* (W9BGP3), *Yersinia pestis* (Q8ZEB3), and *Serratia marcescens* (A0A656VNU6). Conserved key residues involved in the chloride ion transport in the ClcA antiporter are highlighted in red. Conserved residues involved in ion selectivity filter in the ClcA antiporter is highlighted in blue. Yellow-highlighted regions represent the conserved sequences within ClcB proteins. Asterisks indicate fully conserved, colons indicate strong conservations (>0.5), and periods indicate weak conservations (= or <0.5). This sequence alignment was analyzed by CLUSTALW. (**B**) Phylogenetic tree of ClcB proteins from the species listed in (**A**). Transmembrane helices are indicated as boxes with numbering (*E. coli* K-12). Phylogenetic tree are visualized using MEGA 11 software; the branch lengths are displayed at the bottom. (**C**) An overall structure of ClcB homodimer. Colored residues in (**A**) are indicated. To distinguish each monomer, one subunit of the homodimer is visualized more transparently.

ClcB (also known as *mriT* and *ynfJ*), a member of the bacterial ClC chloride channel family, has crucial roles in controlling intracellular chloride ion concentration and enhancing cell viability in the extremely acidic condition. Although *clcB* has not been studied as thoroughly as *clcA*, a double knock-out mutant of *clcA* and *clcB* exhibited a much lower survival in acidic conditions than wild-type and single knock-out mutants [14]. ClcB is also conserved within gram-negative bacteria and is considered as a chloride ion transporter (Fig. 4). As previously discussed, *V. cholerae* is extremely sensitive to pH 2.5 [5], probably due to lacking XAR system. Whether the absence of ClcB in *V. cholerae* could contribute to extreme sensitivity to pH 2.5 needs to be addressed.

Analyzing the homologues of ClcA and ClcB suggests that ClcB contains key residues similar to those found in ClcA. Because the glutamate gate in ClcB is located similarly to those in ClcA, the selectivity filter and chloride binding sites in ClcB appear to function in a similar manner to those in ClcA. Further research is required to determine the precise mechanism of ClcB in chloride transport.

## Conclusions

In bacteria, acid resistance systems including XAR and ATR have been commonly used as survival strategies in extracellular acidic environments such as the human gastrointestinal tract. Among the AR systems, AR2, AR3, AR4, and AR5 are classified as amino acid-mediated AR systems. In the amino acid-mediated AR systems, amino acid antiporter, amino acid decarboxylase, and ClC chloride antiporter are three major components. Amino acid antiporters transport negative charged substrates (Glu, Arg, Lys, and Orn) into the cytoplasm and export positively charged products (GABA, Agm, Cad, and Put), respectively. Amino acid decarboxylases consume protons generated by the influx of HCl as they convert amino acids into positively charged products. Finally, ClC chloride antiporters prevent inner membrane hyperpolarization by transporting chloride ions to the extracellular solution. In this process, the membrane potential of neutralophilic bacteria changes from negative inside to positive charge inside, resulting in an electrochemical gradient similar to acidophiles. This characteristic resolves electrophysiological problems caused by excess intracellular protons generated in extremely acidic conditions.

The ClC chloride antiporter plays a significant role in the amino acid-mediated AR system. This transporters are found in a wide range of organisms, ranging from bacteria to humans, and its key residues are highly conserved. Prokaryotic *clcA* genes encode ClC transporters that control the AR system in acidic conditions. In the ClC antiporter, key residues in the ion selectivity filter are two glutamate residues that coordinate chloride ion binding in the center of the ClC antiporter. This selectivity filter releases two chloride ions and binds to one proton from the outside simultaneously. This process promotes survival of bacteria from acidic conditions by preventing membrane hyperpolarization, thus allowing bacteria to colonize the human intestinal tract.

## Figures and Tables

**Table 1:** Summary of the substrates, products, and proteins utilized in amino acid-mediated acid resistance systems in *E. coli*.

	AR2	AR3	AR4	AR5
Substrate	Glutamate	Arginine	Lysine	Ornithine
Amino acid antiporter	GadC	AdiC	CadB	PotE
Amino acid decarboxylase	GadA/B	AdiA	CadA	SpeF
Product	γ-aminobutyrate (GABA)	Agmatine	Cadaverine	Putrescine
